# Early symptoms of bone and soft tissue sarcomas: could they be diagnosed earlier?

**DOI:** 10.1308/003588412X13373405386213

**Published:** 2012-09

**Authors:** Mike Maguire

## Abstract

**George A, Grimer R** Early symptoms of bone and soft tissue sarcomas: could they be diagnosed earlier? *Ann R Coll Surg Engl* 2012; **94**: 261–266 doi: 10.1308/003588412X13171221590016

The thrust of the above article seems to be that GP ‘awareness and referral of sarcomas remain poor’ to specialist centres and it suggests‘amendments to current guidelines and clearer referral pathways’ and ‘robust education strategies’ for GPs. To put this in context of primary care: given that there are 2,700 cases per year in the UK in a population of 50 million adults, an average GP list size of 1,800 patients and 90% of cases presenting to the GP, a rough estimate is that a GP would expect to see a sarcoma every 11.4 years or 2–3 cases in his or her working life. I also note that the peak incidence is the eighth decade for soft tissue sarcomas and seventh decade for bone sarcomas.

Regarding the methodology, it is not clear from what origin the information came to complete ‘a semi-structured proforma’, which included the essential information about when the patient presented to the GP initially and what the initial symptoms were. It is hoped that this was gleaned from the contemporaneous GP notes accessed through the GPs themselves, which I feel would carry some significance. However, information gathered purely from patient recollections in hindsight following the diagnosis and probable (painful) treatment would have low accuracy.

Despite the apparent delay in referral to a specialist centre there is no discussion as to why this is, apart from a comment about ‘lack of awareness’. This study has no evidence to support such a statement. The authors could have discussed the limitations of the diagnostic criteria by which individual symptoms are likely to have very low specificity (with the exception of a mass) in patients in their 60s and 70s who commonly have painful arthritides. Diagnosis is not clinched by specialist centres but by accurately reported imaging, the access for which is variable for GPs.

Once a diagnosis is made the pathways are very clear through the two-week wait system. Do the authors feel that the explicit pressure on GPs to refer less to specialist centres through financial penalties if GPs refer too much might be a contributing factor? I am unsure whether the authors are aware of the national cancer strategy for England (January 2011), which incorporates health promotion, encouraging earlier presentation, more screening and better access to GPs for diagnostic tests, and the use of cancer risk scores (from the QResearch database or the Risk Assessment Tool), which identified symptoms or coded variables in primary care to improve cancer outcomes.

